# Influence of Laser Irradiation Settings, during Diode-Assisted Endodontics, on the Intraradicular Adhesion of Self-Etch and Self-Curing Luting Cement during Restoration—An Ex Vivo Study

**DOI:** 10.3390/ma15072531

**Published:** 2022-03-30

**Authors:** Eleftherios Terry R. Farmakis, Franziska Beer, Ioannis Tzoutzas, Christoph Kurzmann, Hassan Ali Shokoohi-Tabrizi, Nikos Pantazis, Andreas Moritz

**Affiliations:** 1 Department of Endodontics, School of Dentistry, National and Kapodistrian University of Athens, 11527 Athens, Greece; 2 Independent Researcher, 1130 Vienna, Austria; dr.beer@beamed.at; 3 Department of Operative Dentistry, School of Dentistry, National and Kapodistrian University of Athens, 11527 Athens, Greece; tzoudent@dent.uoa.gr; 4 Clinical Division of Conservative Dentistry and Periodontology, University Clinic of Dentistry, Medical University of Vienna, 1090 Vienna, Austria; christoph.kurzmann@meduniwien.ac.at (C.K.); hassan.shokoohi-tabrizi@meduniwien.ac.at (H.A.S.-T.); andreas.moritz@meduniwien.ac.at (A.M.); 5 Department of Hygiene, Epidemiology and Medical Statistics, School of Medicine, National and Kapodistrian University of Athens, 15772 Athens, Greece; npantaz@med.uoa.gr

**Keywords:** laser, laser-assisted endodontics, diode-assisted endodontics, adhesion, self-etch cement, self-curing cement, intraradicular post, polymer post, endodontics, restoration, glass fiber post, push-out test

## Abstract

Background: Diode-assisted endodontics is nowadays utilized for pulp space disinfection, but little is known on the bonding potential of this lased root dentin when the tooth is restored with an intracanal polymer post. Objectives: to investigate the influence of diode laser irradiation settings, in laser-assisted endodontics, on the intraradicular bonding of composite materials. Methods: Sixteen two-rooted, maxillary first premolars were collected, prepared up to F4 (Protaper Universal. Dentsply-Maillefer, Ballaigues, Switzerland), and randomly assigned in two groups: group A (chopped mode or short pulse), diode irradiated according to protocol, pulse 25 ms, power 2.5 W, and group B (microchopped mode or ultrashort pulse), pulse 25 μs, peak power 12 W (both groups GentleRay. KaVo Dental, Biberach an der Riss, Germany). Buccal canals were irradiated, palatal ones served as controls. Canals were then obturated, post space was created in all canals, and quartz-fiber posts (ICE light Danville. Danville Materials, San Ramon, CA, USA) were cemented by self-etch self-curing cement (Max Cem Elite. Kerr, West Collins Orange, CA, USA) (Max Cem Elite. Kerr, Brea, CA, USA). A week later, teeth were sectioned horizontally in 1 mm increments. Push-out test was conducted in a Zwick testing machine (Zwick Roell, Ulm, Germany) at 1 mm/min speed, and the force required to dislodge the post from each specimen (F-max) was recorded. Weibull regression models were applied for statistical analyses. Results: Differences in F-max by group (control vs. chopped mode vs. microchopped mode) and height (meaning the apical-to-coronal position of each specimen along the root) were statistically significant (*p* < 0.05 in all cases). Conclusions: Short pulses (or chopped mode) had a profound positive effect on the quality of intraradicular bonding, while Ultrashort pulses (or microchopped mode) affected it negatively. In addition, apically positioned bonding proved weaker compared with more coronally located specimens.

## 1. Introduction

Bacterial invasion of the pulp space is the main causative factor of apical periodontitis [1]. Effective elimination of the bacterial population in this confined space is essential for a successful outcome of endodontic treatment [2].

Laser irradiation has been suggested for pulp space disinfection since 1986 [3]. One of the main advantages of root canal irradiation, as advocated, is their bactericidal action in deeper dentin layers. Bacteria can penetrate 1 mm into the dentinal tubules [4], and conventional irrigants (without activation) act as deep as 100 μm [5] vs. 500 μm for diode [6] and 1 mm for Nd:YAG irradiation, respectively [7].

Many wavelengths, different parameters, and either sole treatments or combined/adjunct approaches have been suggested, with varying reported success of bacterial reduction [8,9,10,11,12,13].

The subsequent clinical step of any endodontically treated tooth is its restoration, which in many cases, demands the use of intraradicular post(s) to support a core build [14,15]. Nowadays, these restorations are mainly performed with composite resin materials, requiring bonding to the dentinal substrate [16].

In the available literature, most of the publications on diode-assisted endodontics (diode laser irradiation of the root canal, following the preparation prior to obturation) investigate their bactericidal potential. Very few papers were found investigating the influence of this irradiation on root dentin alteration (mainly collagen alteration due to heat accumulation) [17,18,19], while others tested the resin-to-coronal dentin bonding [20,21,22]. No paper was located discussing the intraradicular adhesion (bonding) of luting cement to endodontically treated then irradiated root canal dentin, as happens in the case of diode-assisted endodontics and subsequent restoration.

In addition, as several pulse parameters have been suggested for diode-assisted root canal sterilization, little is known on the efficacy of each pulse profile concerning the safety of the patient (temperature increase on the external root surface) and their bactericidal capacity [23,24].

The null hypothesis was that diode laser irradiation settings will have no effect on the intraradicular bonding of composite luting cement following diode-assisted pulp space disinfection, especially as the trepan (during post preparation) will remove a significant layer of superficial root canal dentin.

## 2. Materials and Methods

### 2.1. Endodontic Preparation

The protocol was approved by the Ethics Committee of the Dental School of the National and Kapodistrian University of Athens, Greece (Ref: 165C on 25/7/2018), and informed consent was released by all patients.

A single experienced operator performed all endodontic procedures and irradiations (blinded concerning the applied laser parameters), and the laser device was maintained and calibrated right before the experiment to ensure correct performance [25].

**Figure 1:**
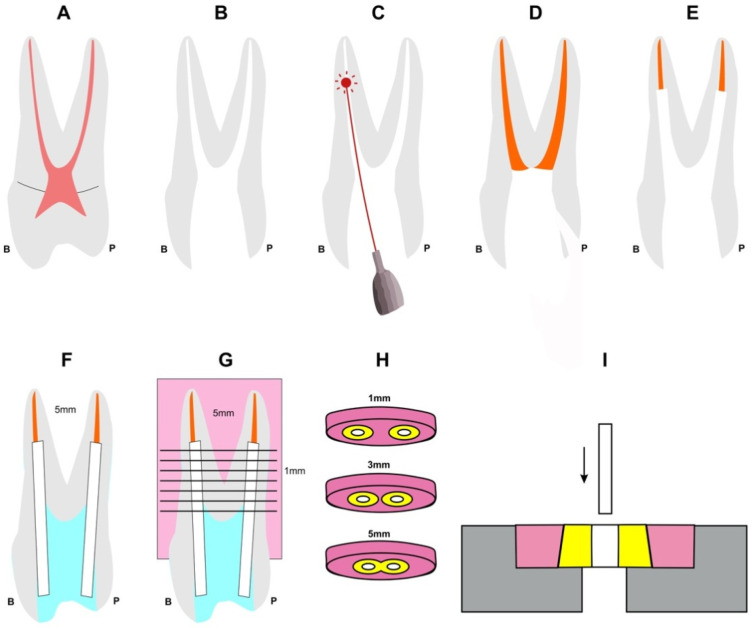
All stages of the experimental procedure. (**A**) sound premolar; (**B**) after cleaning and shaping; (**C**) diode irradiation of the buccal canal; (**D**) endodontic obturation with gutta percha and epoxy resin sealer; (**E**) post space preparation; (**F**) post cementation and restoration of the opening access; (**G**) tooth embedded in epoxy resin and sectioned; (**H**) samples to be tested; (**I**) push-out test performed to an apical-to-coronal direction.

Eighteen sound, two-rooted upper first premolars (with fully formed apices), extracted for orthodontic reasons from young adults up to 30 years old, were selected. The length of selected teeth was in the range of 20 ± 1 mm (Figure 1A).

Immediately after extraction, teeth were cleaned in an ultrasonic bath with 3% hydrogen peroxide and stored in normal saline solution (0.9% NaCl) at 5 °C until use. Then, access cavity on the occlusal surface of all teeth was performed via an Endo Access Bur (Dentsply Maillefer, Ballaigues, Switzerland), and the borders of the cavity were smoothened with an Endo Z bur (Dentsply Maillefer, Ballaigues, Switzerland) with a high-speed handpiece under water spray. Root canals were located, verified for accessibility with a No8 K-File (Dentsply Maillefer, Ballaigues, Switzerland), and each canal orifice was enlarged with Gates-Glidden burs (#1, #2 and #3), at a depth of 2 mm below the anatomical neck of the tooth, and the working length was determined as the total length of each root, minus 1 mm.

Next, chemomechanical preparation of all root canals was performed utilizing the ProTaper system (Dentsply Maillefer, Ballaigues, Switzerland) up to the F4 instrument (ISO 040 tip size, a 6% apical third taper, and then a progressively decreasing taper in the coronal portion of the file), with simultaneous use of EDTA gel 18.6% (File Eze. Ultradent, South Jordan, UT, USA) and irrigation with 2.5% NaOCl solution (2.5 mL between each file and 10 mL final rinsing) (Figure 1B).

### 2.2. Diode Irradiation

After the completion of the chemomechanical preparation, the teeth were randomly organized into two equal groups of 8 teeth (Groups A and B), while the remaining two were used in the pilot studies (no laser irradiation applied on those). The buccal root canals of each experimental group were irradiated with a diode laser (GentleRay 980 nm Diode Laser, KaVo Dental GmbH, Biberach an der Riss, Germany) according to designated parameters: Group A, (8 samples) in the chopped mode setting, with a 200 nm diameter fiber, a pulse duration of 25 ms, a mid-stop of 25 ms, at a power of 2.5 W (referenced in the paper either as short pulse or chopped mode), and Group B, (8 samples) in the microchopped mode setting, with a 200 nm diameter fiber, a pulse duration of 25 μs, average power 2.5 W and peak power 12 W (discussed in the paper either as ultrashort pulse or microchopped mode, as named by the manufacturer). The palatal served as control.

**Figure 2:**
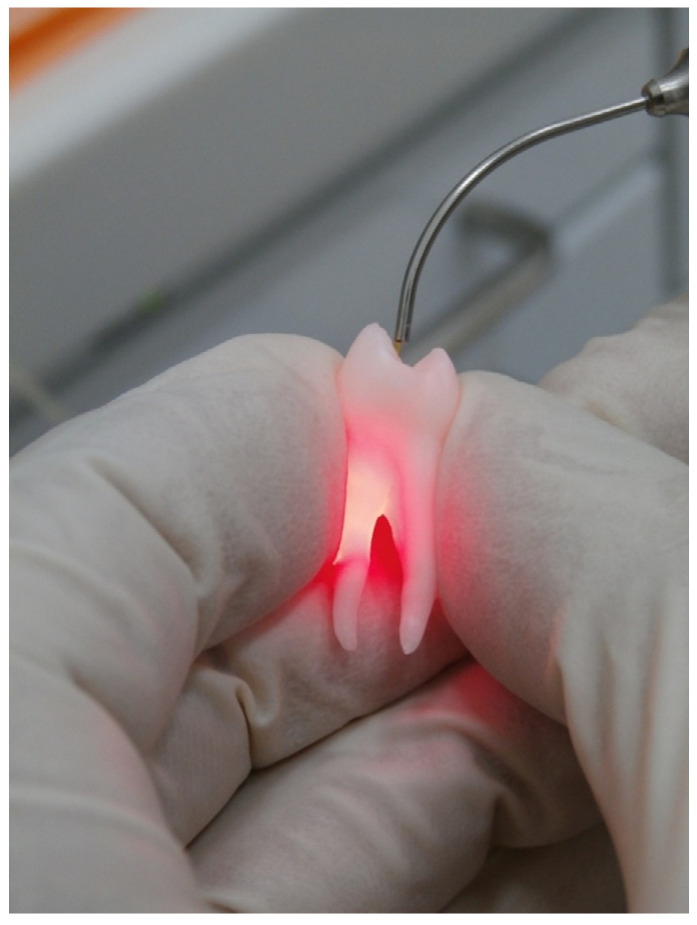
Diode irradiation of the buccal canal.

Irradiation protocol consisted of six irradiation cycles of 5 s, each 30 s apart, to allow thermal relaxation of the root canal dentin. Spiral motion of the optical fiber was applied from the tip to the orifice entrance and completed in 5 s. During irradiation, the root canals were filled with sterile saline. In both experimental groups, the palatal canal was not irradiated (Figure 1C and Figure 2). A single experienced operator (blinded concerning the applied parameters) performed all endodontic procedures irradiations, and the laser device was maintained and calibrated right before the performance of the experiment.

Then, all root canals were filled with ISO No40 Gutta Percha points and epoxy resin sealer (AH-26, Dentsply De Trey GmbH, Konstanz, Germany), using the cold lateral condensation technique, and the excess filling material was removed at the canal orifice with a hot instrument. A sterile cotton pellet was placed in the pulp chamber, the access opening was sealed with a temporary filling (Cavit-G, 3 M ESPE, St. Paul, MN, USA), then stored in 37 °C and 100% humidity for a week, to ensure the setting of the sealer (Figure 1D).

After a week, gutta-percha was removed at a length of 15 ± 1 mm, so that in all samples, a 5-mm obturation remained intact. This was performed with the help of Gates Glidden and PEESO burs, and then the accompanying taper-less drill of the designated glass fiber resin-reinforced posts was applied in the canal (ICE light Danville, Danville Materials, San Ramon, CA, USA), red trepan, the diameter of 1.4 mm, no taper). Then, ultrasonication was performed in each canal for 20 s (EMS, Nyon, Switzerland), followed by 10 mL sterile saline irrigation via a 25 G needle to remove the created debris, and then dried with paper points (Figure 1E). The post space was inspected by a dental microscope (Pico, Zeiss, Oberkochen, Germany) to verify the absence of gutta-percha remnants.

Posts were cut to a 12 mm length by a diamond disc under water cooling to ensure they would not stick out of the occlusal surface but instead stay embedded in the pulp chamber during final coronal restoration. The prepared post spaces were filled with self-etching, self-curing resin cement (Max Cem Elite (Kerr, West Collins Orange, CA, USA)), using the automix intracanal tips in combination with a spiral Lentulo to minimize air entraption. Cement was also applied on the posts, and they were immediately placed in the full well length.

The occlusal opening was sealed with the conventional resin bonding system (UniEtch-Bisco, Schaumburg, IL, USA, Optibond solo and Herculite XRV-Kerr, West Collins Orange, CA, USA), 10 min later, to ensure complete setting of the resin cement (according to the manufacturer) (Figure 1F). Then, all teeth were embedded in cylindrical silicon matrices (of 16 mm diameter) filled with hard transparent autocuring epoxy resin (EpoFix kit, Struers, Ballerup, Denmark), prepared according to manufacturer’s instructions, along their long axis, and left undisturbed for 48 h in order to let the resin set fully.

Next, the roots were sectioned along their longitudinal axis, with the use of a microtome (Isomet 11-1180, Buehler Ltd., Evanston, IL, USA) in 1 mm increments (Figure 1G), starting 5 mm from the apex (6 slices in total—the first one being 1 mm from the apical tip of the post, and the sixth being at the orifice entrance; Figure 1H, showing representative slices of 1, 3 and 5 mm). All samples were then measured with a digital caliper to ensure the exact 1 mm thickness of each slice (to an accuracy of 0.01 mm) and individually stored in distilled water until subjected to the push-out test.

During the cutting process, some specimens (from both groups) were destroyed, leaving 29 specimens from Group A and 23 from Group B intact (and 29 + 23 = 52 controls).

#### 2.2.1. Push-Out Test

**Figure 3:**
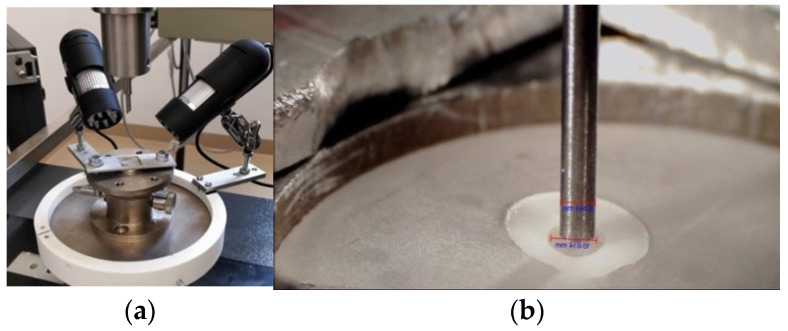
(**a**) Custom-made sample holder with the 2 digital microscopes; (**b**) Piston pressing the post.

The push-out tests were carried out with the help of a Zwick testing machine (Zwick Roell, Ulm, Germany). For this purpose, a specially created design and a custom-made sample holder were used (Figure 3a). The sample holder had a flat metallic surface with a hole of 1.8 mm, slightly larger than the diameter of the specimen to be tested. With this sample-holder construction, it was possible to adjust the piston (0.8 mm diameter) exactly to the center of the sliced post. (Figure 3b). The piston had a flat end and was slightly smaller than the posts’ diameter (1.1 mm) and was precisely positioned under the guidance of two digital microscopes (SVBONY SV606 Digital Microscope, 50–1000 × USB Microscope, SVBONY, Hongkong, China), set up to a 90-degree angle to each other, via video monitoring. The digital microscopes were also used to visually document (record) the test. The piston moved with 1 mm/min in a coronal direction and started with a starting load of 1 N. (Figure 1I). The maximum force applied for the dislodging to occur (F-max) was recorded for each specimen.

#### 2.2.2. SEM Investigation

Following a failure, all specimens were examined under ESEM (TM1000, Toshiba, Tokyo, Japan) to determine the type of failure (cohesive/adhesive/mixed).

#### 2.2.3. Statistical Analysis

Differences in the force required to dislodge the post from the root canal walls (F-max) by group (Control vs. Short Pulses vs. Ultrashort pulses) and height (meaning the apical-to-coronal position of each slice along the root) were investigated using Mann–Whitney *U* tests, Wilcoxon matched-pairs signed-rank tests, and Weibull regression models with shared frailty term, to account for potential correlation between repeated measurements on the same specimen ID (same tooth, same level on both roots, identical composition of dentin). Exploratory analysis revealed nonlinear trends of F-max by height; thus, Weibull regression models also included a quadratic height term.

Data preparation and analysis have been performed using Stata (StataCorp. 2015. Stata Statistical Software: Release 14. StataCorp, College Station, TX, USA), and *p*-values < 0.05 were considered as indicating statistical significance.

## 3. Results

In only three specimens, the cement was unevenly distributed around the post (air entraption).

As the drill was taper-less, and the internal bonding surface of the root canal was cylindrical and identical in all specimens, the values displayed herein are both in Newtons (N) and in Megapascals (MPa), connected by the following equation:

S = T/A

where S is the shear bond strength, T is the tension (F-max) applied, and A is the bonding area. (A = 2πrh, where r is the radius of the canal, and h its height).

The F-max recordings between the controls of Groups A and B were very similar (median 29.8 N vs. 30.7 N (or 3.38 MPa vs. 3.49 MPa) *p* = 0.531), proving the validity of the model.

From the push-out test results, it seems that under the influence of radiation, the irradiated samples as a whole group showed higher F-max recordings than the Control (nonlased) samples by 9 N or 1.02 MPa (median), although the difference was not statistically significant

**Figure 4:**
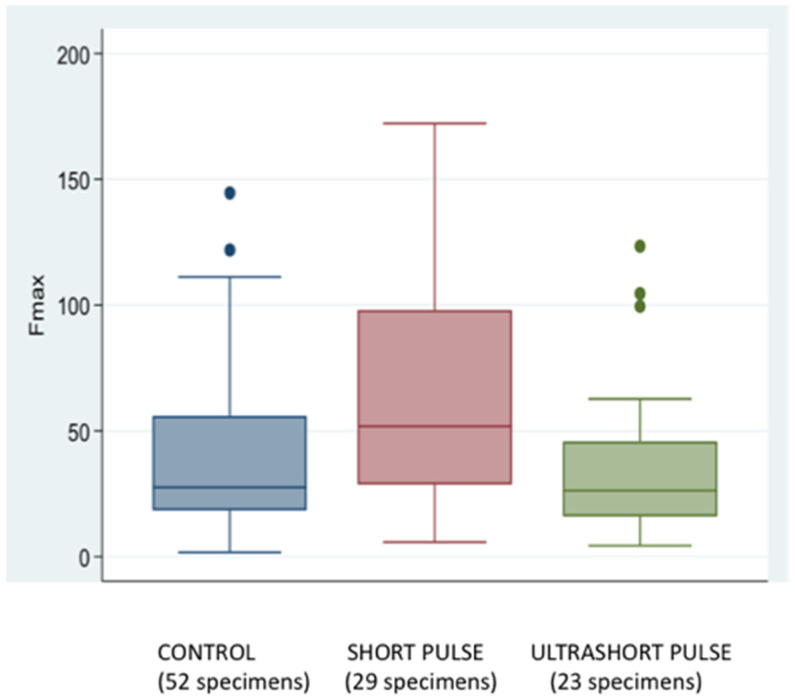
Box plot of F-max by the group.

However, when the individual groups and their results were compared, the A group (short pulse) presented statistically significantly higher forces (22.9 N or 2.60 MPa on median) recorded on the push-out test than the control group (*p*-value = 0.004) (Figure 4). Additionally, the A group showed statistically significantly higher forces recorded to the push-out test than group B (ultrashort pulse) (50.9 N vs. 26.5 N, or 5.78 MPa vs. 3.01 MPa, *p* = 0.003).

In contrast, Group B produced results slightly lower than the control group (26.5 N vs. 30.7 N or 3.01 MPa vs. 3.49 MPa, *p*-value = 0.434), with no statistical significance. The differences between groups A and B compared with the control group were more pronounced in group A (22.9 N or 2.60 Mpa) than in group B (−2.9 N or 0.32 MPa), and the difference in performance between the two groups was statistically significant (*p* = 0.007). (The minus symbol refers to the median differences of F-max recordings (Group B performed inferior to the Control) within the interquartile range of recordings (IQR)).

**Figure 5:**
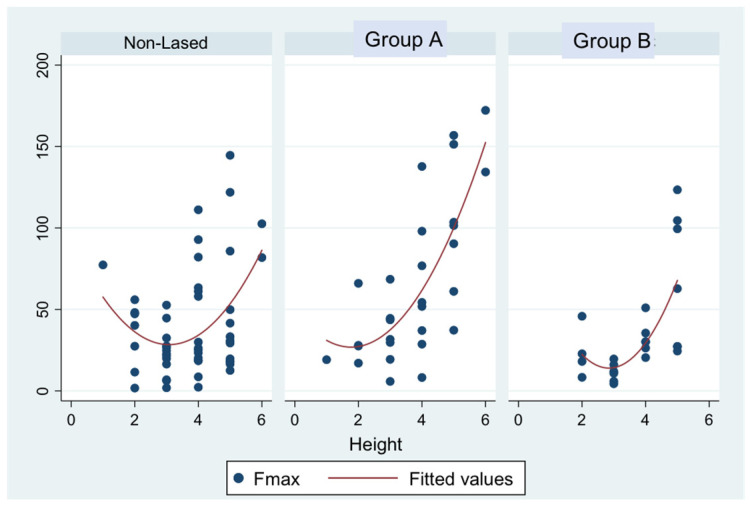
Scatterplots of F-max vs. height by the group with quadratic fitted values.

**Figure 6:**
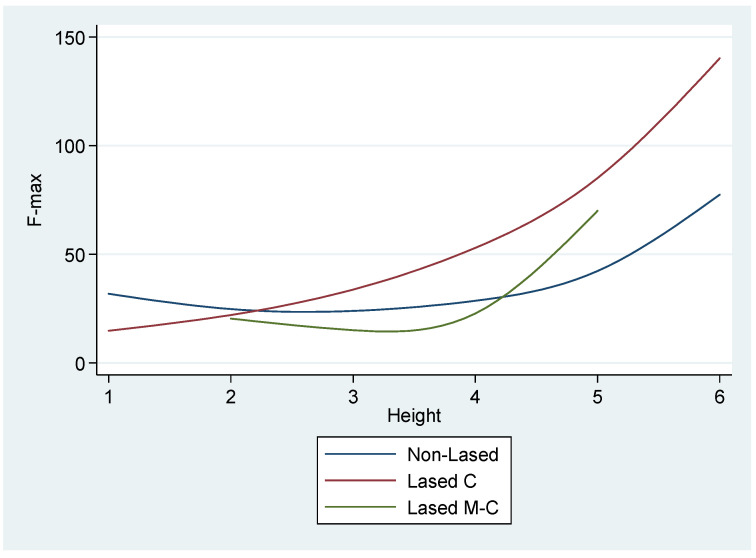
Predicted median F-max by height and group; results from Weibull models fitted separately to each group.

The F-max recordings in the lased specimens were also related to the position of the tested specimen along the root length (*p* < 0.001), a phenomenon which remained significant in all groups (Figure 5) but with the shape of the relation being significantly different across groups (interaction *p*-value = 0.026, Figure 6).

### SEM Results

**Figure 7:**
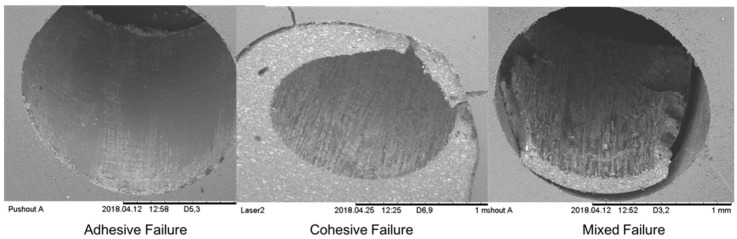
Failure types.

**Figure 8:**
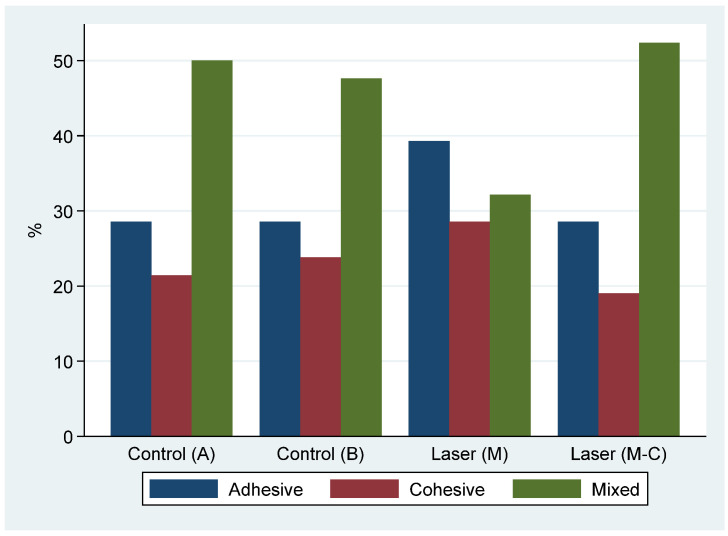
Distribution of failure type by the group.

The distribution of failure type (adhesive, cohesive, mixed, Figure 7) was similar in the two control groups (*p* > 0.999); thus, they were combined into one. The distribution of failure type did not differ significantly between the two laser and combined control groups (*p* = 0.611). Finally, there were no significant differences in the distribution of failure type between the two laser groups (*p* = 0.410) nor between a laser combined group and the combined control group (*p* = 0.708) (Figure 8).

## 4. Discussion

The success of the endodontic treatment depends on the effective elimination of the intracanal bacterial population, but the endodontic treatment itself is actually completed when a final restoration is performed upon the tooth. In many cases, this final step may require the placement of an intracanal (intraradicularly) retained post and core [14]. The endodontically treated residual dentin (following chemomechanical preparation) is far from being an ideal bonding substrate due to its chemical alterations and makes it prone to failures during the final intracanal restoration [26].

Furthermore, any intervention that may further affect the dentin, and subsequently the bonding quality, should be thoroughly checked—and in this case, it was the diode 980 nm radiation during laser-assisted endodontics that additionally affected the endo-treated intraradicular dentin [17,18,19].

The use of diode laser technology in endodontic therapy has been widely studied. Although the wavelengths of these lasers (805–980 nm) are not fully adapted to the individual components of dental tissues (poor to moderate absorption by hydroxyapatite, water, dentin, and enamel) [27], it applies a form of energy, which has broad dental indications [8,24,28,29,30,31,32]. Additional advantages of these lasers are that they are price affordable and not bulky as devices.

It is well documented that the wavelength, the different parameters of laser devices and fibers used, the output power, and the duration and manner of the application of irradiation have different effects on the disinfection of root canals [8,33,34].

Diode lasers have been shown to exhibit satisfactory bactericidal effects on relatively deep layers of dentin (up to 500 microns) during the laser-assisted endodontic treatment [6], although the actual penetration depth for diode irradiation is about 4 cm in dentin because dentin’s optical properties, favor scattering and transmission rather than absorption [35,36]. The suggested setting when diode laser irradiation is applied for laser-assisted endodontics is 2.5 W for short pulses [23,37].

The common belief for the antimicrobial action of lasers was the absorption of radiation from microbial cells—either direct absorption or through refraction, due to the hydroxyapatite crystals of dentin [38].

The indirect (refractive/diffusive) action was used in the past to explain the death of microbial cells through different dentine thicknesses in laboratory experiments [6,7,33,38]; the antimicrobial activity of various laser radiation lengths has been confirmed by several in vivo studies [8,39,40,41,42,43,44], but also by systematic reviews [45,46].

However, it was the work of Pirnat and colleagues in 2011 [47] that showed that the existence of chromophores in the microbial environment is a prerequisite condition for the absorption and bactericidal activity of radiation. Otherwise, this action is due to the increase in the temperature of the dentin, with everything that entails for the change in its composition and whatever associated possible consequences—from patient safety to periodontal ligament safety, to possible dentin damage.

This ex vivo study investigated whether the manufacturer’s proposed settings for diode-assisted endodontics are suitable, in terms of the subsequent restoration of these laser-assisted endodontically treated teeth, with resinous systems and polymer posts.

For the standardization of the parameters of this experiment (and to avoid unnecessary variations and bias), the model of the first upper double-rooted premolar was chosen. With this decision, the substrate (dentin) would have an identical composition in both groups (lased and control), in all comparable levels along the root, as it forms at the same time [48].

Dentin is reported to possess a low thermal conductivity, ranging from 0.363 and 0.666 Wm^−1^K^−1^; thus, one is expecting that most dentin collagen alteration will occur within a fraction of the canal wall thickness. Another influential factor affecting thermal conductivity is porosity. Porosity is more profound following endodontic treatment, resulting in further reduction in the already low thermal conductivity of the treated dentin [49]. It is likely that different pulses and radiation parameters are associated with different changes in the substrate of dentin [17,18,19].

Laser irradiation (through temperature increase) causes either contraction of collagen (when low levels of energy are applied) or denaturation or destruction (in higher ones), depending on the energy absorbed at a specific point. Collagen is denatured (coagulated) at 60 °C, thermally decomposed (vaporized) at temperatures above 100 °C, and then carbonized at temperatures above 150 °C [50,51].

Thus, in the deeper layers of dentin, during the course of temperature rise (around 60–70 °C), hydrolysis of the heat-labile cross-links occurs, while the maintenance of heat-stable cross-links is responsible for the residual tension within collagen fibrils. Additionally, under heating, swelling and shrinkage of collagen fibrils follow the unwinding of the triple helix with the maintenance of heat-stable intermolecular cross-links [52,53].

According to the aforementioned studies, the diode bactericidal effect was expected to be expressed superficially along the canal wall, and the removal of that affected layer from the post’s drill would reveal unaffected dentin. In the current experimental setting, at 5 mm short of the working length, the prepared root canal is expected to be 650 μm wide. Thus, the drill (which was 1400 μm diameter) is estimated to have removed 750 μm circumfencially at that level, and around 575 μm at the CEJ (orifice of the canal), according to the taper of the prepared canal. The results of this study indicate that the action of the diode irradiation is still profound in the deeper layers of dentin that are exposed by the trepan.

Since the results of a previous report [24] showed that the greatest deviation in the various settings was between the short and the ultrashort pulse of 2.5 W (same average power, but significantly different peak power, 2.5 W vs. 12 W), it was decided to perform the bonding and subsequent push-out test utilizing these two pulses. Additionally, it was decided that the root canals were to be obturated by lateral condensation of cold gutta-percha and sealer so that there would be no additional thermal stress on the root dentin [54] and no systematic error (bias) in the outcome.

Due to the intact crown and the length of the post space, it was decided to use a spiral lentulo in order to place timely, a sufficient amount of cement to the apical end of the well and to avoid air entraption in its mass, especially at the level of the remaining gutta-percha [55,56]. The examination of the specimens revealed just three specimens showing air entraption, proving the validity of the approach. Furthermore, the taper-less design of the trepan also ensured an identical, cylindrically shaped bonding area for all specimens.

From the results obtained herein, it was concluded that the position of the specimen along the root did affect the results. The more coronally the specimen examined, the higher the force needed to dislodge, revealing a transition in behavior, from root dentin “evolving” to coronal dentin (different type of collagen and increased number and width of dentinal tubules). This finding is consistent with another paper [57].

The surface destruction of collagen is confirmed by the associated surface increase in the microhardness of dentin [58]. What the literature does not adequately explain is the increased hardness in deeper layers. This increase in microhardness may be due to the fact that the collagen of radical (root) dentin has a larger diameter (compared with the crown dentin) and a different orientation [59], with a possible interpretation that as the diode energy reaches the deeper layers of radical dentin, causes contraction of the local collagen, resulting in increased microhardness in deep dentin layers. Additionally, a cross-linking phenomenon of the denaturized triple collagen helix (occurring during thermal relaxation of the dentin) cannot be excluded [60,61,62,63].

Still, the heat transfer during laser irradiation on tooth layers and the prediction of the temperature profile in the tooth structure is not well defined, but analyses and trends tend to be more realistic if adopting a non-Fourier model [64].

In this research, a logical assumption would suggest that these two different pulses would not affect the dentin much differently, as they both increase the external root temperature statistically differently but within safety limits [24]. The results point to a totally different direction, and the interpretation lies in the lasers’ physics: the increased power is not due to an actual increase in energy but from the decrease in the duration of the pulse. Therefore, during the spiral motion of the optical fiber, the ultrashort pulse is not so often emitted, so, although much stronger, it radiates fewer points (covering a smaller surface) of the radical dentin during the spiral motion of the fiber. Additionally, as the conductivity of dentin is initially low and, due to the increase in the endodontic treatment porosity, decreases even more, the energy remains focused in an extremely confined point and does not sufficiently diffuse to the surrounding dentin.

Thus, the short pulse is assumed to perform a more uniform distribution of energy on the surface of intracanal dentin due to the comparatively longer pulse duration. On the other hand, ultrashort, high-intensity pulse lasers (micro or femtosecond pulses) can ablate material only superficially, without causing significant thermal damage in deeper layers [65].

What this experiment showed is that short pulse diode 980 nm laser irradiation, following the chemomechanical preparation and prior to the obturation of the root canal, had a positive effect on the bonding of a self-etching, self-curing system on the radical dentin. Although the F-max recordings were higher in the irradiated group, no statistically significant difference was observed. A significantly positive difference was observed between the control group and the short pulse group. The interpretation can be attributed to the changes of the remaining collagen of endodontically treated dentin under the influence of short pulse diode radiation, both superficially and in deeper layers.

In a few cases, a direct drop out of the restorative material was observed, revealing, once more, the unpredictability of a robust hybrid layer formation in the root dentin compared with coronal dentin. The unfavorable C-factor of the round root canal during the setting of the resinous material should also be taken into consideration when discussing these issues [66]. Summarizing all the above, it is impossible to expect a perfect bonded interface because of the histology of root dentin (especially when it has undergone such detrimental treatments), the shrinkage of resinous cement, and the sensitivity of the bonding technique itself [67].

As regards the interpretation of the difference in the behavior of the two pulses, the ultrashort pulse is expected to cause greater local damage superficially to collagen, confirmed by the thermal results of Beer et al. [24]. Furthermore, it should be taken into account that chemically treated human dentin has a higher porosity and, at the same time, bears less specific thermal conductivity and thermal diffusion compared with healthy dentin [49].

All the aforementioned leads to the conclusion that the ultrashort pulse, due to its high power and small duration, has an extreme deteriorating, local (superficial) effect and does not affect the deepest layers, as the short pulse. This is also supported by observations of other researchers [68].

It is worth mentioning that other researchers have used the diode irradiation but following post space preparation [69,70,71]. The results are not comparable, as this ex vivo scenario examined the influence of laser irradiation in laser-assisted endodontics and its subsequent restoration, not laser-assisted prosthodontics.

Moreover, a project evaluating the bactericidal effect of these pulses would aid the knowledge in the field of laser-assisted endodontics and laser-assisted dentistry.

Another limitation of this study was that the push-out test was performed immediately without any thermocycling, aging, or any other fatigue. Therefore, further analysis would be useful in order to evaluate the influence of aging/mastication forces/temperature alternation on the bond strength of the cement to dentin. The same applies to the type of cement that was applied herein. A different cement might have ended up with different results.

Another thing that is important to note is that during the literature research, apart from the group of Beer and coworkers that use a particular diode device in different fields and sets of experiments, no other set of projects has been detected, investigating diode laser-assisted “restorative” dentistry. Using the same device is important for the observed results of various experiments that cover a range of clinical applications and successor actions (e.g., endodontics followed by restorative dentistry and prosthodontics), so the results will have a strong correlation between them due to the identical characteristics of applied radiation (pulse duration, pulse intensity, output power, etc.).

## 5. Conclusions

Within the limitations of this ex vivo study, it can be concluded that the settings of the laser irradiation during diode-assisted endodontics affect the retention of fiber posts, adhered via intracanal with self-etching self-setting resin cement. Short pulses (of the 980 nm wavelength) increased the retention, whereas the ultrashort pulses resulted in a negative effect. More studies are needed to design predictable outcome protocols for laser-assisted dentistry.

## Data Availability

Not applicable.
